# Clinical Incorporation of the 2017 Classification of Periodontal Diseased Conditions: Part I (Diagnosis of Periodontitis Involving Data From 4,993 Patients)

**DOI:** 10.7759/cureus.63423

**Published:** 2024-06-28

**Authors:** Priyanka T G, Ashwini Athul, Harini D, Shiba Thasneem, Nasser I, Cyril Benedict, Anitha Vijayarangan, Ashwath Balachandran, Shanmugam Muthukali

**Affiliations:** 1 Periodontics, Chettinad Dental College and Research Institute, Chennai, IND; 2 Public Health Dentistry, Chettinad Dental College and Research Institute, Chennai, IND

**Keywords:** peri-implantitis, chronic gingivitis, dental diagnosis, classification, periodontitis

## Abstract

Background

The new classification system for periodontal and peri-implant diseases mentioned a few key points, including (1) “clinical gingival health," which was defined for the first time, and (2) staging and grading system. Thus, the present study defines the practicability of using the current classification system in day-to-day practice.

Aim

The primary aim of this study is to determine the effectiveness of the new classification system for periodontal and peri-implant diseases and its application in routine dental practices.

Materials and methods

This is a cross-sectional retrospective, non-interventional study consisting of 4,993 patients who were under active periodontal therapy as well as supportive periodontal therapy.

Results

Among patients diagnosed with dental biofilm-induced gingivitis with no underlying risk factors, 1105 (73.3%) exhibited bleeding on probing and 897 (45%) showed supragingival or subgingival calculus. Among the stage II grade B periodontitis group, 488 (28.9%) showed beginning destructive periodontal disease and 197 (50.4%) showed established destructive periodontal disease.

Conclusion

The implementation of the new classification system in routine dental practice has been readily accepted by clinicians. Staging and grading system of classification helps in assessing the severity, extent, and progression of disease.

## Introduction

The 2017 World Workshop classification for periodontal and peri-implant diseases integrates the biological and clinical research knowledge that have become evident since the 1999 International Classification of Periodontal Diseases [1]. The essence of this classification system was to include the severity, extent, and rate of progression of the periodontal disease, as well as susceptibility of patients to periodontal diseases, thereby determining a definition for periodontal health [2]. The main focus of the 2017 World Workshop classification in the field of periodontology was to adopt a reductionist model that can be practically implemented in routine dental practice where more than 95% cases of periodontal disease are diagnosed and managed [3,4].

The 2017 classification system primarily includes staging and grading of periodontal diseases. The staging of periodontal disease is based on the severity of disease presentation and the complexity of the disease. Grading reflects the susceptibility of individuals to periodontal disease as well as the risk determinants that are responsible for clinical attachment loss (CAL) and bone loss [1,5]. After establishment of staging and grading of the periodontal disease, the previous history of any periodontal disease or periodontal treatment needs to be considered for further treatment planning. Evaluation of the bleeding on probing (BOP), periodontal pocket depth, and radiographic alveolar bone loss helps in determining the current health status of the patients.

The 2017 classification system has also withdrawn the distinction between chronic and aggressive periodontitis as there were not ma biological studies to support both of these conditions as separate entities. The 2017 classification system acknowledged the necrotizing periodontal diseases and periodontitis as a manifestation of systemic disease as separate entities [5].

The two main propositions of the 2017 classification are as follows: the earliest explanation of clinical gingival health and the determination of working diagnosis of the periodontal health status for each patient. Thus, it helps in better clinician-patient understanding and cooperation to establish personalized treatment and oral hygiene care to achieve well-defined therapeutic outcomes [1-2,6-7].

The primary objective of this study is to determine the effectiveness of the 2017 World Workshop classification for periodontal and peri-implant diseases and its application in routine dental practices.

## Materials and methods

Study design

This is a cross-sectional retrospective, non-interventional study of the patients who had visited the Chettinad Dental College and Research Institute, Kelambakkam, Chennai, Tamil Nadu, India. Data were collected from previous patient records who visited the Department of Periodontology, Chettinad Dental College and Research Institute, for either active periodontal therapy or supportive periodontal care. The patients were diagnosed on the basis of the new 2017 World Workshop classification system.

Sample size calculation

After performing a goodness-of-fit test using sample sizes from comparable research, the minimum sample size needed for this investigation was determined to be 4,800, for which the study’s power was 0.95. As a result, we included 200 samples, which was more than the minimum number needed to improve the study’s validity. The statistical analysis was performed using SPSS Version 20 (IBM Corp., Armonk, NY).

Study population and selection

The present study was approved by the Institutional Human Ethics Committee (IHEC), Chettinad Academy of Research and Education (IHEC - II/0305/23). A single dental practitioner from the Department of Periodontology at the Chettinad Dental College and Research Institute collected all the participant data retrospectively using the Excel software (Microsoft Corp., Redmond, WA).

A total of 4,993 patients were included in this study from the previous patient records, which included patients in active periodontal therapy and supportive periodontal therapy. The Department of Public Health Dentistry at Chettinad Dental College and Research Institute calculated the sample size and analyzed and interpreted the data.

Sampling technique

The convenience sampling method was used where suitable cases requiring active and supportive periodontal therapy were selected from previous patient records over a specific period of time between October 2019 and April 2024.

Study duration

Data were collected from the patients who visited the Department of Periodontology, Chettinad Dental College and Research Institute, from October 2019 to April 2024.

Inclusion criteria

Male and female patients aged 18 to 55 years, patients who are in need of active and supportive periodontal therapy, and patients with no previous history of periodontitis were included in the study.

Exclusion criteria

Patients who were below 18 years and pregnant/lactating patients were excluded from the study.

Statistical analysis

The statistical analysis was performed using SPSS Version 20 (IBM Corp., Armonk, NY). Descriptive statistical analysis was represented by a frequency distribution table. The correlation between different clinical parameters was assessed using one-way ANOVA and the chi-square test.

## Results

**Figure 1:**
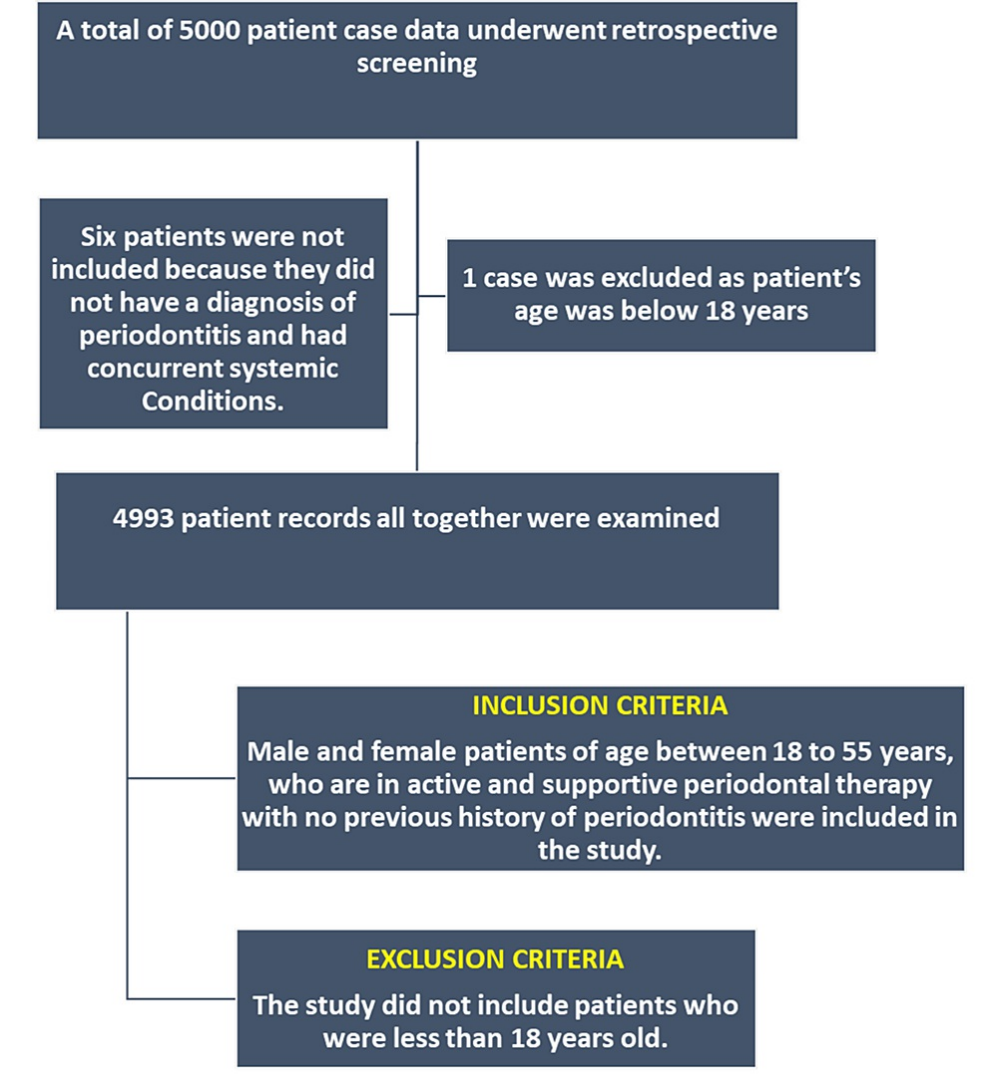
Study design

A total of 4,993 patients were included in the study, with 2,715 (54.4%) males and 2,278 (45.6%) females. Six patients were not included in the study because the diagnosis was not of periodontitis and they were diagnosed with systemic conditions. One patient was excluded as the age was below 18 years (Figure 1).

When the correlation between probing pocket depth (PPD) and periodontal screening and recording (PSR) index were assessed, it revealed that the PPD was significantly higher among patients with deep pockets (4.58 ± 1.94) followed by those with furcation defects (4.06 ± 0.78), with a p-value of 0.001 (Table 1).

**Table 1:** Correlation between periodontal screening and recording index and probing pocket depth *Statistically significant results (p < 0.05) PSR, periodontal screening and recording

PSR	Mean	Standard Deviation	95% Confidence Interval for Mean		P-value
			Lower Bound	Upper Bound	
Healthy	2.2983	.67985	2.1498	2.4467	0.001*
Bleeding after probing	2.5598	11.37742	1.9849	3.1347	
Supra/subgingival calculus	2.4184	.62265	2.3910	2.4457	
Shallow pocket	2.7232	.56387	2.6874	2.7591	
Deep pocket	4.5859	1.94222	4.4044	4.7675	
Furcation	4.0067	.78763	3.5705	4.4428	

The assessment of correlation between OHI-S score (oral hygiene index - simplified) and PPD/CAL score revealed that no significant association was found between OHI-S and PPD or CAL in the study.

**Figure 2:**
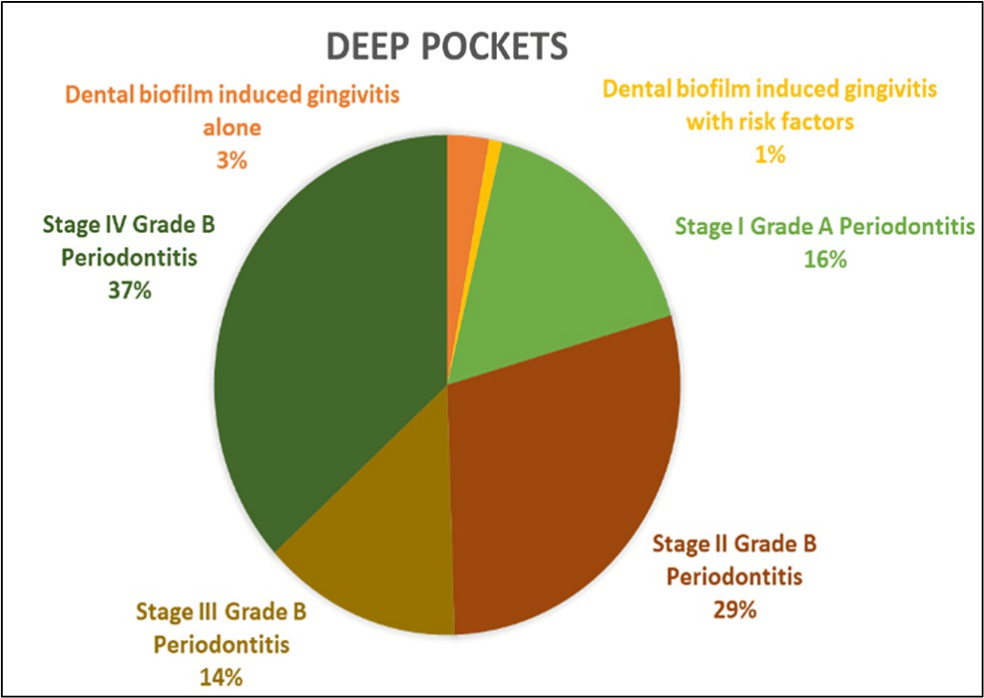
The percentage distribution of probing pocket depth (deep pockets > 7 mm) among different periodontal diagnostic groups

**Figure 3:**
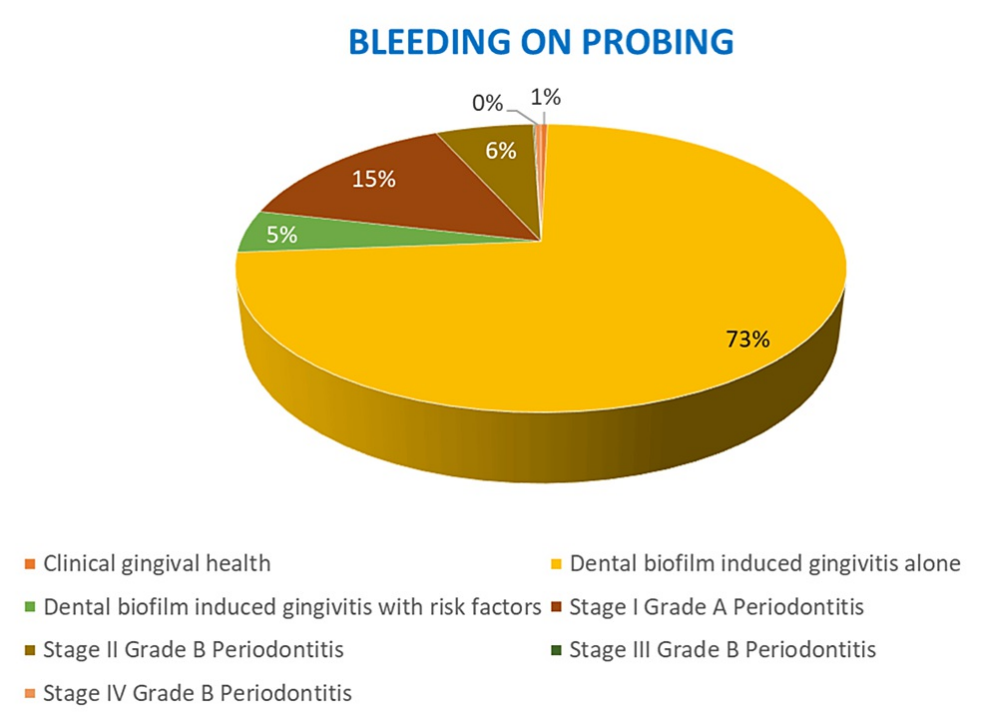
The percentage distribution of bleeding on probing among the different periodontal diagnostic groups

The correlation between PSR and diagnosis revealed that among patients diagnosed with dental biofilm induced gingivitis with no underlying risk factors, 1,105 (73.3%) exhibited BOP (Figure 2) and 897 (45%) showed supragingival/subgingival calculus. These findings were significant with a p-value of 0.001 (Figure 3).

Among the patients diagnosed with stage II grade B periodontitis, 301 (31.6%) had shallow pockets and 128 (29%) had deep pockets. These results were found to be statistically significant, with a p-value of 0.001. The assessment of deep pockets and furcation among different groups revealed the highest percentage recorded in the stage IV grade B Periodontitis group with 162 (36.7%) and 14 (93.3%), respectively. This was statistically significant with a p-value of 0.001 (Table 2).

**Table 2:** Correlation between PSR index and periodontal diagnosis *Statistically significant results (p < 0.05) DBIG, dental biofilm induced gingivitis; PSR, periodontal screening and recording; %, percentage of distribution in each group; n, number of patients in each group

PSR	Diagnosis	n	%
Healthy	DBIG - biofilm alone	61	73.5
	DBIG - risk factors	4	4.8
	Stage 1 - grade A	16	19.3
	Stage 2 - grade B periodontitis	2	2.4
Bleeding after probing	Clinical gingival health	6	0.4
	DBIG - biofilm alone	1,105	73.3
	DBIG - risk factors	73	4.8
	Stage 1 - grade A	222	14.7
	Stage 2 - grade B periodontitis	94	6.2
	Stage 3 - grade with periodontitis	1	0.1
	Stage 4 - grade B periodontitis	6	0.4
Supra/subgingival calculus	Clinical gingival health	5	0.3
	DBIG - biofilm alone	897	45.0
	DBIG - risk factors	84	4.2
	Stage 1 - Grade A	412	20.7
	Stage 2 - grade B periodontitis	507	25.4
	Stage 3 - grade with periodontitis	4	0.2
	Stage 4 - grade B periodontitis	85	4.3
Shallow pocket	DBIG - biofilm alone	135	14.2
	DBIG - risk factors	7	0.7
	Stage 1 - grade A	289	30.3
	Stage 2 - grade B periodontitis	301	31.6
	Stage 4 - grade B periodontitis	221	23.2
Deep pocket	DBIG - biofilm alone	13	2.9
	DBIG - risk factors	4	0.9
	Stage 1 - grade A	74	16.7
	Stage 2 - grade B periodontitis	128	29.0
	Stage 3 - grade with periodontitis	61	13.8
	Stage 4 - grade B periodontitis	162	36.7
Furcation	DBIG - biofilm alone	1	6.7
	Stage 4 - grade B periodontitis	14	93.3
P-value			0.001*

Assessing the correlation between Russell’s periodontal index and diagnosis, patients with dental biofilm induced gingivitis showed 407 (41.7%) clinically normal gingiva and 1,449 (75.8%) had simple gingivitis. Among patients with Stage II grade B periodontitis, 488 (28.9%) showed beginning destructive periodontal disease, whereas 197 (50.4%) showed established destructive periodontal disease. These results were statistically significant with a p-value of 0.001. 22 (73.3%) of patients with Stage IV grade B periodontitis showed terminal disease, and this was statistically significant with a p-value of 0.001 (Table 3).

**Table 3:** Correlation between Russell’s periodontal index and diagnosis *Statistically significant results (p < 0.05) DBIG, dental biofilm induced gingivitis; PSR, periodontal screening and recording; %, percentage of distribution in each group; n, number of patients in each group

Rusell’s Periodontal Index	Diagnosis	n	%
Clinically normal	Clinical gingival health	10	1.0
	DBIG - biofilm alone	407	41.7
	DBIG - risk factors	84	8.6
	Stage 1 - grade A	296	30.3
	Stage 2 grade B periodontitis	166	17.0
	Stage 3 grade with periodontitis	6	0.6
	Stage 4 grade B periodontitis	7	0.7
Simple gingivitis	Clinical gingival health	1	0.1
	DBIG - biofilm alone	1,449	75.8
	DBIG - risk factors	70	3.7
	Stage 1 - grade A	191	10.0
	Stage 2 - grade B periodontitis	177	9.3
	Stage 3 - grade with periodontitis	2	0.1
	Stage 4 - grade B periodontitis	21	1.1
Beginning destructive periodontal disease	DBIG - biofilm alone	339	20.1
	DBIG - risk factors	18	1.1
	Stage 1 - grade A	471	27.9
	Stage 2 - grade B periodontitis	488	28.9
	Stage 3 - grade with periodontitis	5	0.3
	Stage 4 - grade B periodontitis	365	21.6
Established destructive periodontal disease	DBIG - biofilm alone	15	3.8
	Stage 1 - grade A	53	13.6
	Stage 2 - grade B periodontitis	197	50.4
	Stage 3 - grade with periodontitis	53	13.6
	Stage 4 - grade B periodontitis	73	18.7
Terminal disease	DBIG - biofilm alone	2	6.7
	Stage 1 - grade A	2	6.7
	Stage 2 - grade B periodontitis	4	13.3
	Stage 4 - grade B Periodontitis	22	73.3
P-value			0.001*

## Discussion

The master stroke of 2017 classification in periodontology relies on its implication in routine dental practices. The extensive application of this 2017 classification was mainly because it was embraced by clinicians readily due to the fact that it takes into consideration various parameters that are consistent. Periodontal parameters that are substantially impacted by the treatment, such as BOP and PPD, cannot be used for evaluating disease stage. This is a key tenet of the staging process, which is to be carried out at the time of the initial evaluation, and, hence, patients cannot fall back to a less severe form of periodontitis following treatment [8]. Grading is intended to reflect the patient’s predisposition to periodontitis as it essentially accommodates all risk factors that have triggered periodontal bone loss caused during the patient’s lifespan [8]. Furthermore, previous history of periodontal disease is one of the most appropriate indicators of future disease presentation [9,10].

For the first time, clinical gingival health is specified in the 2017 classification. Studies reveal that between 50% and 90% of adults have gingivitis at varying degrees [11,12]. When evaluating periodontal tissues, the diagnosis of “clinical gingival health” indicates a paradigm shift. This renders it possible to establish clearly defined outcomes for treatment at the patient level.

In the current investigation, the majority of patients had stage II grade B periodontitis in accordance with the analysis of patient distribution. This was consistent with the results of Raza et al. [13], who found that most stage II grade B cases were represented in the patient distribution. As opposed to this, Graetz et al. [14] discovered that the majority of patients had stage III grade C periodontitis.

In the present study, the correlation between PPD and PSR was found to be statistically significant. This was in accordance with a study by Khocht et al. [15], in which the efficacy of PSR index was compared to radiographs for periodontal disease screening. According to the current study, less than 6 mm (shallow pockets) of probing depth is evident in nearly 31.5% of individuals with stage II grade B periodontitis. This was also consistent with the results of Covington et al. [16], who reported that adult periodontitis was present in around 30% of cases with a probing depth of 4 mm or more. Another study revealed that 75.1% of adult Japanese people had attachment loss of 3 mm or more [17], which was contradictory to the results of the present study.

Our investigation confirmed Khocht et al.'s [15] observation that PSR scores of 3 and 4 are the most prevalent PSR scores among periodontitis patients. Given that approximately 95% of individuals have periodontal disease and show signs of gingival inflammation, PSR scores of 3 and 4 should be common, as predicted by the National Health and Nutrition Examination Survey (NHANES) data and examination of periodontal disease prevalence in our patient sample [18,19].

The current study revealed that there was a significant correlation between PSR index and periodontal diagnosis. These findings were similar to a study by Primal et al. [20], which concluded that patients with PSR index score of less than 3 were diagnosed with dental biofilm induced gingivitis. There was a significant correlation found between PSR index and deep pockets, which is in agreement with the results reported by Primal et al. [20], who mentioned that with higher scores of PSR index, PPD and attachment loss were greater. As determined by the present study, PSR index was a moderately good indicator of periodontal disease, and this was in accordance with the results reported by Primal et al. [20].

Landry et al. [21] reported that the periodontal disease severity was not accurately estimated by PSR index as it does not measure the epithelial attachment, which is an indirect measure of CAL that occurred on the root surface. This was contradictory to the findings of the present study, which revealed a significant association between PSR index and CAL.

Study limitation

This study took data directly from previous patient records to test the adaptability, acceptance, and practicality in implementing the new 2017 classification in general clinical practices. Also, the patient follow-up was done for a limited number of years, thereby increasing the chances of exclusion of refractory periodontal cases. Further studies on the staging and grading system of periodontal diseases are required to aid in determining the accuracy of this current classification system.

## Conclusions

The prime highlights of this new classification system were the inclusion of the clinical gingival health, the staging and grading pattern of defining the periodontal diseases, and conditions taking into account the rate of periodontal destruction, thereby defining the future periodontal disease occurrence and progression. This system also marks the first step toward the application of personalized medicine principles to the treatment of periodontitis. Additionally, it offers the structure required for the incorporation of biomarkers into prognosis and diagnosis.

Thus, the study highlights the widespread use and acceptance of the new classification system in regular dental practices, which aids in easy communication, better treatment planning by taking into account the risk factors for future periodontal breakdown, and cumulative treatment planning to render the best possible clinical outcomes to the patients. Repetitive use of this new classification system gives an overview of its effectiveness in diagnosing periodontal diseases and conditions.
